# Gut microbiota-driven immunomodulation in tuberculosis: targeting dysbiosis to overcome drug resistance

**DOI:** 10.3389/fimmu.2026.1889315

**Published:** 2026-08-07

**Authors:** Qi Nie, Xujuan Hu, Yingjie Zhang, Ling Pan, Xiaoqing Zhang, Yong Zhou, Tingting Fu, Lijuan Zheng, Fan Xiao, Yuan Liu, Si Du, Jieguang Liu, Leyiyi Liu, Xianglian Xiong, Yuanjun Wu, Dan Fan, Lixuan Tao, Fuli Ren

**Affiliations:** 1 Wuhan Jinyintan Hospital, Tongji Medical College of Huazhong University of Science and Technology, Wuhan, Hubei, China; 2 Department of Multidrug-resistant/Drug-resistant Tuberculosis (MDR/DR-TB), Wuhan Jinyintan Hospital, Tongji Medical College of Huazhong University of Science and Technology, Wuhan, Hubei, China; 3 Clinical Biospecimen Resource Center, Wuhan Jinyintan Hospital, Tongji Medical College of Huazhong University of Science and Technology, Wuhan, Hubei, China; 4 Emergency Department, Puren Hospital, Wuhan University of Science and Technology, Wuhan, Hubei, China; 5 State Key Laboratory for Diagnosis and Treatment of Severe Zoonotic Infectious Diseases, Wuhan, Hubei, China

**Keywords:** anti-TB therapy, DR-TB, dysbiosis, FMT, gut microbiota, probiotics, tuberculosis

## Abstract

Tuberculosis (TB) remains a major global health threat, hampered by the escalating prevalence of multidrug-resistant (MDR) and extensively drug-resistant (XDR) strains. Managing these resistant infections demands protracted, intricate, and frequently hepatotoxic drug regimens with poor efficacy, toxicity, and adherence issues. This landscape underscores an urgent need to move beyond a purely antimicrobial-focused paradigm. Converging lines of evidence now firmly position the gut microbiota—a pivotal orchestrator of systemic immunity, metabolic homeostasis, and drug metabolism—as a central determinant in both TB pathogenesis and therapeutic success. Our review describes a vicious cycle at the heart of contemporary TB management. A critical and often overlooked trigger is the profound and persistent gut microbiota dysbiosis induced by the anti-TB medications themselves. Far from an incidental side effect, this dysbiosis is strongly implicated as a pathological driver in preclinical models and observational studies. It undermines pulmonary host defense through the gut-lung axis, aggravates anti-tuberculosis drug-induced liver injury (ATB-DILI), and cultivates a systemic state of chronic inflammation coupled with metabolic dysregulation (such as impaired lipid metabolism). Paradoxically, this host environment fosters Mycobacterium tuberculosis persistence and disease progression. In drug-resistant TB, this vicious cycle is amplified, is associated with therapeutic failure and may contribute to the selection of resistance in correlative studies. To disrupt this cycle, we assess the translational promise of interventions targeting the microbial ecosystem. This encompasses a multi-pronged strategy: employing probiotics, prebiotics, and tailored dietary modifications to restore ecological balance; utilizing fecal microbiota transplantation (FMT) for more profound restoration; and pioneering the development of novel, narrow-spectrum antimicrobials designed to preserve commensal flora. We contend that the integration of microbiome stewardship into TB care is an indispensable evolution in our approach. By concurrently targeting the pathogen and fortifying the host’s intrinsic microbial defenses, this holistic strategy presents a transformative pathway to surmount drug resistance, alleviate treatment-related toxicity, and improve patient outcomes.

## 1 Introduction

Tuberculosis (TB) continues to impose a staggering global health and economic burden, with an estimated 10 million new cases and 1.5 million deaths annually (1). This persistent crisis is dramatically intensified by the emergence and spread of multidrug-resistant (MDR-TB) and extensively drug-resistant (XDR-TB) strains, which render conventional, prolonged antibiotic regimens increasingly ineffective and toxic (2). To overcome this impasse, a paradigm shift beyond the traditional pathogen-centric view is urgently needed.

The gut microbiota, a vast and dynamic ecosystem of microorganisms, is now recognized as a master regulator of host immunity, metabolism, and drug pharmacology (3). Dysbiosis, the disruption of this ecosystem, is implicated in a wide array of diseases. In the context of TB, compelling evidence reveals that gut microbiota composition critically influences susceptibility to *Mycobacterium tuberculosis* (*Mtb*) infection and the efficacy of anti-TB therapy (4). Crucially, the standard anti-TB drugs themselves induce profound and lasting dysbiosis, creating a detrimental cycle: impaired gut barrier function and altered microbial metabolites compromise systemic immune responses, exacerbate drug-induced hepatotoxicity, and may even foster an environment conducive to the selection of drug-resistant strains (5, 6). This recognition of dysbiosis as a central mediator positions gut microbiota disruption not merely as a side effect, but as a central pathological nexus linking treatment failure, adverse outcomes, and drug resistance. This review will first dissect the immunomodulatory mechanisms of the gut-lung axis and the consequences of therapy-induced dysbiosis, before focusing on its specific role in DR-TB. We then critically evaluate microbiota-targeted interventions, and finally, explore the novel frontier of the gut-brain axis in TB patient mental health.

This review synthesizes recent advances to systematically delineate the multifaceted role of the gut microbiota in TB pathogenesis and drug resistance. We first dissect the immunomodulatory mechanisms of the gut-lung axis and the consequences of therapy-induced dysbiosis. We then critically evaluate the structural and metabolic alterations of the gut microbiome in DR-TB. Furthermore, we assess the translational potential of microbiota-targeted interventions—including probiotics, FMT, and microbiome-informed drug development—as strategies to restore homeostasis, enhance treatment efficacy, and mitigate adverse effects. Finally, we explore the underappreciated impact of the gut-brain axis on the mental health of TB patients. By integrating perspectives from microbiology, immunology, and clinical medicine, this review aims to chart a path toward innovative, holistic therapeutic strategies that co-manage the pathogen and the host’s microbiome to ultimately overcome the enduring challenge of TB.

## 2 Gut microbiota and its immune regulatory mechanisms in tuberculosis

### 2.1 The gut-lung axis: immunomodulatory mechanisms in tuberculosis

The gut microbiota is a critical modulator of host immunity, with particular relevance to TB. Through the gut-lung axis, this microbial community exerts remote control over pulmonary immune responses, thereby shaping host defense against *Mtb*. Gut microbiota composition directly influences the activation and differentiation of CD4+ T cells, a cell population essential for controlling *Mtb* infection (7). Gut-derived metabolites, including short-chain fatty acids (SCFAs) and tryptophan derivatives, support immune homeostasis (8). Among these, the microbial metabolism of tryptophan generates bioactive compounds that activate the aryl hydrocarbon receptor (AhR), a signaling pathway that modulates immune responses and helps sustain immune equilibrium within both the gastrointestinal tract and the systemic compartment (9).

The dynamic interaction between gut microbiota and immune cells encompasses a complex network of signaling pathways. Gut microbiota-derived metabolites enhance the ability of immune cells to detect and respond to pathogens by influencing the expression of pattern recognition receptors (PRRs), which is crucial for initiating appropriate immune responses to *Mtb*. Furthermore, the gut microbiota influences the adaptive immune response by modulating the differentiation of T helper cells into various subsets, including Th1 and Th17 cells, which are critical for effective anti-tubercular immunity (10, 11). Th17 cells—key players in mucosal immunity—are particularly susceptible to gut microbiota influence. Dysbiosis induces an imbalance in Th17 cell activation, thereby contributing to the pathogenesis of TB. The production of interleukin-17 (IL-17) by Th17 cells has been linked to enhanced recruitment of neutrophils and macrophages to the site of infection, facilitating the clearance of *Mtb* (12).

**Figure 1:**
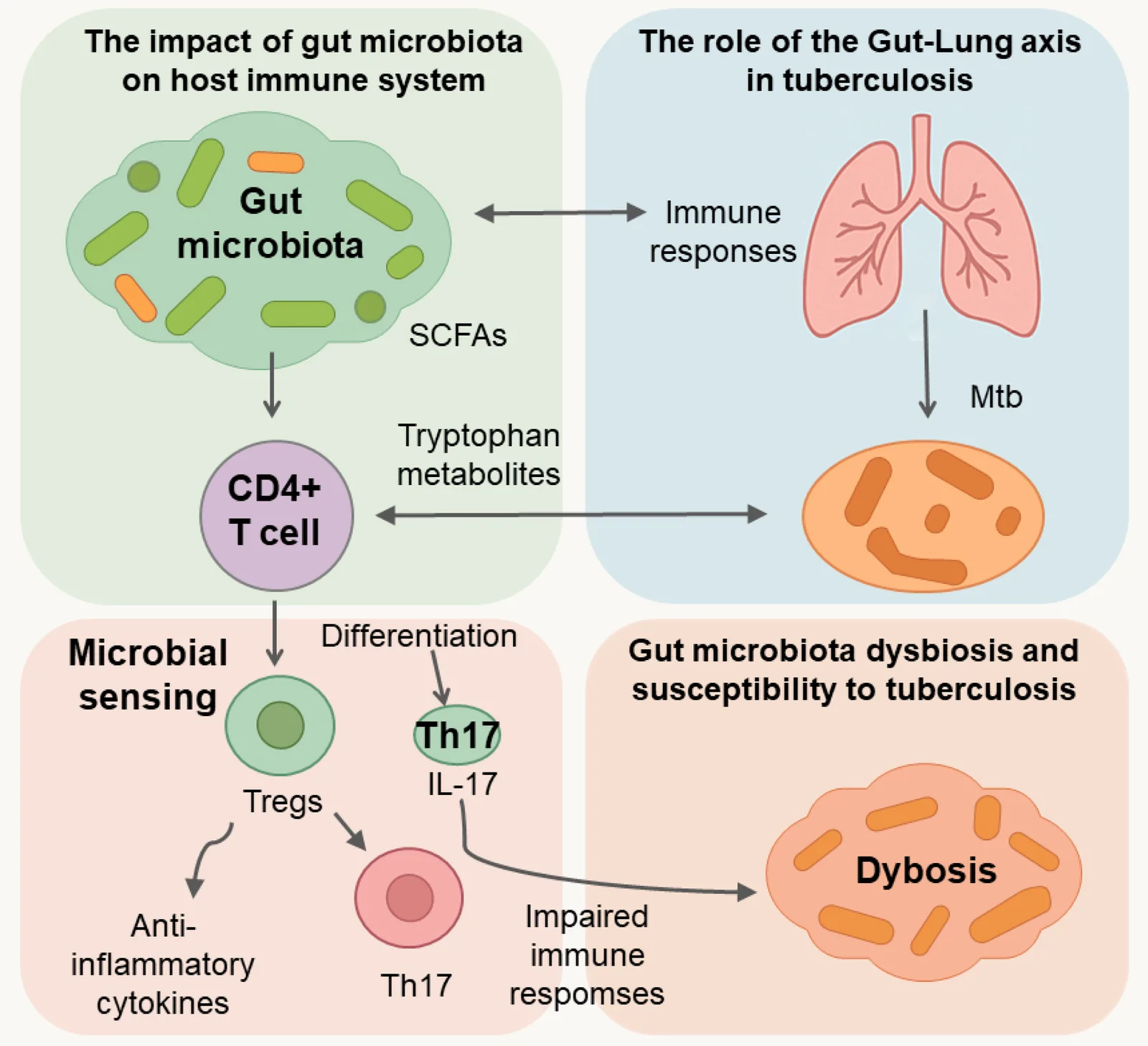
The impact of gut microbiota on immune responses and tuberculosis susceptibility. This diagram illustrates the intricate relationship between gut microbiota, immune responses, and tuberculosis susceptibility. The gut microbiota influences host immunity by modulating CD4+ T cell activity, including the differentiation of Th17 and Tregs. Dysbiosis in the gut microbiota, can impair immune responses, potentially increasing vulnerability to *Mtb* infection through altered immune system dynamics.

Dysbiosis, whether induced by malnutrition, comorbidities like obesity, or antibiotic therapy, disrupts this axis, skewing lung immune responses towards a hyperinflammatory state that paradoxically favors *Mtb* infection and disease progression (13, 14). As shown in **Figure 1**, anti-TB therapy–induced dysbiosis exemplifies this disruption, creating a vicious cycle that impairs lung immunity, aggravates hepatotoxicity, and promotes drug resistance. Dysbiosis impairs immune responses, characterized by diminished cytokine production and altered T cell populations essential for controlling *Mtb* infection (10, 11). Furthermore, the gut and lung microbiota engage in critical bidirectional communication during *Mtb* infection. Alterations in the gut microbiome due to antibiotic treatments can lead to dysbiosis, which not only affects gut health but also compromises lung immunity, making the host more susceptible to *Mtb* colonization (15–17). The presence of certain gut bacteria has also been associated with enhanced expression of protective long non-coding RNAs (lncRNAs) that promote anti-TB immunity (18).

In summary, the gut-lung axis is a central regulator of pulmonary anti-mycobacterial immunity. Its dysregulation establishes a permissive environment for *Mtb*, linking distal intestinal health directly to lung disease outcomes (19, 20). Consequently, strategies restoring gut microbiota balance, such as probiotics, FMT, or dietary interventions, represent a promising therapeutic avenue to enhance anti-TB immunity and improve patient outcomes.

### 2.2 Gut microbiota dysbiosis and susceptibility to tuberculosis

A diverse and balanced gut microbiota is a cornerstone of systemic immune competence. In TB, this balance is critically breached. Patients with active disease exhibit a characteristic gut dysbiosis marked by loss of protective commensals and expansion of pro-inflammatory pathobionts. This section details how this specific dysbiotic signature not only reflects disease activity but actively contributes to heightened susceptibility and compromised host defense against *Mtb*.

Research has identified specific alterations in the gut microbiota structure of tuberculosis patients, which may contribute to their heightened susceptibility to the disease. In patients with active TB, there is a marked enrichment of opportunistic pathogens and a depletion of beneficial commensal bacteria. For instance, a comparative study revealed that the gut microbiota of TB patients exhibited a substantial increase in genera such as *Escherichia* and *Shigella*, while beneficial taxa like *Lactobacillus* and *Bifidobacterium* were significantly reduced (21). These compositional shifts not only reflect dysbiosis but also suggest a mechanism through which altered gut microbiota impact systemic inflammation and immune responses. It is important to note that the presence of certain pathogenic bacteria may exacerbate inflammatory pathways, leading to a more severe clinical presentation of TB. Furthermore, a dysbiotic gut microbiome is associated with impaired immune function, as evidenced by reduced production of key anti-mycobacterial cytokines (22). These distinct microbial signatures in TB patients highlight the potential of gut microbiota profiles as biomarkers for susceptibility and as therapeutic targets to restore balance and enhance immunity.

However, these associations are subject to significant confounding. Factors such as concurrent antibiotic exposure for other infections, nutritional status—which independently shapes the microbiome—geographic location, which affects both microbiome composition and *Mtb* strain diversity, and underlying comorbidities including HIV infection or diabetes are rarely fully controlled for in human studies. Therefore, the independent contribution of gut dysbiosis to TB susceptibility remains to be definitively established.

## 3 The impact of anti-tuberculosis drugs on gut microbiota and its clinical consequences

### 3.1 Characteristics of gut microbiota dysbiosis induced by anti-tuberculosis drugs

The impact of first-line anti-tuberculosis drugs, particularly the combination of isoniazid, rifampicin, pyrazinamide, and ethambutol (HRZE), on gut microbiota diversity and functionality is of critical importance. These medications are imperative in the treatment of TB, but their administration has been associated with substantial alterations in the gut microbiome. HRZE therapy is associated with the utilization of HRZE and a decline in microbial diversity, a hallmark of dysbiosis, which can lead to a decrease in beneficial bacteria, such as those in the genera *Bifidobacterium* and *Lactobacillus*, while promoting the growth of potentially harmful bacteria. This dysbiotic shift can, in turn, exacerbate gastrointestinal symptoms and compromise overall health (23). Moreover, the metabolic functions of the gut microbiota, including the production of SCFAs that play a crucial role in maintaining gut health and immune function, may also be adversely affected. The dysbiotic state induced by HRZE leads to increased intestinal permeability, inflammation, and altered immune responses, which can complicate TB treatment and recovery (24). Therefore, a comprehensive understanding of these changes is essential for the development of effective strategies to mitigate the adverse effects of anti-TB therapy on gut health.

In the context of MDR-TB, the long-term changes in gut microbiota during treatment are of particular concern. The treatment regimens for MDR-TB frequently entail the administration of more potent and toxic medications, which can further disrupt the gut microbiome. Patients undergoing MDR-TB treatment experience sustained dysbiosis, characterized by persistent alterations in gut microbial composition even after the cessation of therapy. This sustained dysbiosis may contribute to complications such as malnutrition and opportunistic infections, which can impair drug pharmacokinetics, delay immune reconstitution, and ultimately hinder treatment success (25). The long-term implications of such dysbiosis underscore the necessity for meticulous monitoring and potential interventions, such as the administration of probiotics or dietary modifications, to restore gut microbiota balance and support the health of patients undergoing MDR-TB treatment. The intricate relationship between anti-TB therapy and gut dysbiosis warrants further investigation to optimize treatment strategies and enhance patient outcomes.

The degree and duration of dysbiosis vary considerably, depending on factors such as the specific drug regimen—whether first-line or MDR-TB therapy—treatment duration, and host genetics. Moreover, the clinical relevance of particular taxonomic shifts, including decreases in *Lactobacillus* or *Bifidobacterium*, is frequently extrapolated from other disease contexts and may not be directly applicable to tuberculosis. The absence of standardized protocols for microbiome sampling and analysis across studies further limits comparability.

### 3.2 Gut microbiota dysbiosis and antituberculosis drug-induced liver injury

The hepatotoxicity of anti-TB drugs is a major clinical setback, and the gut-liver axis serves as a crucial mediator of this adverse effect. Drug-induced gut dysbiosis compromises intestinal barrier integrity, facilitating the translocation of microbial products that ignite hepatic inflammation and oxidative stress. Here, we dissect this cascade and highlight the emerging role of gut microbiota modulation as a strategic avenue to mitigate ATB-DILI.

Probiotics, particularly strains such as *B. fragilis 839*, have emerged as promising therapeutic agents in the context of drug-induced liver injury, particularly in patients undergoing antituberculosis therapy. The mechanisms by which *B. fragilis 839* exerts its hepatoprotective effects are multifactorial. One of the primary mechanisms involves the restoration of gut microbiota diversity, which is often compromised during antituberculosis treatment. By re-establishing a balanced microbial community, probiotics can enhance the integrity of the intestinal barrier, thereby reducing intestinal permeability and preventing the translocation of harmful substances, such as endotoxins, into the bloodstream. Reducing systemic inflammation is crucial, as elevated levels of inflammatory cytokines contribute to the pathogenesis of drug-induced liver injury. Furthermore, *B. fragilis 839* has been observed to produce metabolites, such as SCFAs, which have been shown to possess anti-inflammatory properties and can promote liver health by modulating immune responses and reducing oxidative stress. A series of experimental studies have demonstrated that the administration of *B. fragilis 839* can significantly lower serum levels of liver enzymes, including alanine aminotransferase (ALT) and aspartate aminotransferase (AST). These enzymes are often used as indicators of liver injury. Furthermore, histopathological examinations revealed improvements in liver architecture, with reduced signs of inflammation and necrosis in treated animals. These findings demonstrate the value of probiotics not only as adjunctive therapies to alleviate ATB-DILI but also as preventive measures to enhance the overall safety and efficacy of anti-TB treatments (13). Future clinical trials are needed to further elucidate the specific pathways involved and establish standardized probiotic interventions in clinical practice.

### 3.3 The impact of gut microbiota dysbiosis on treatment outcomes and drug resistance

The gut microbiota plays a crucial role in the efficacy of various pharmacological treatments, particularly in the context of TB and its drug-resistant forms. In a murine model, antibiotic-induced dysbiosis was shown to reduce the efficacy of isoniazid (INH), a first-line anti-TB medication, by compromising host immune responses and impairing the clearance of *Mtb* (16, 18, 26). Specifically, antibiotic-induced dysbiosis has been demonstrated to alter the composition of the gut microbiota, resulting in an increased abundance of opportunistic pathogens and a concomitant decrease in beneficial bacteria such as *Lactobacillus* and *Bifidobacterium*. This compositional shift not only compromise the body’s intrinsic immune response but also to trigger an escalation in inflammation and tissue damage, consequently intensifying the pathology associated with TB and impeding the therapeutic efficacy of INH (26). Furthermore, the dysbiosis observed in patients undergoing anti-TB therapy has been observed to correlate with a higher incidence of treatment failure in clinical cohorts, although confounding factors remain, highlighting the intricate relationship between gut health and treatment outcomes.

Mounting evidence suggests a correlation between alterations in the composition of the gut microbiota and the emergence of DR-TB strains. The prolonged use of antibiotics, particularly in the treatment of MDR-TB, can lead to significant changes in the gut microbiome, thereby fostering an environment conducive to the emergence of resistant strains. This consequence is of particular concern, as the altered gut microbiota may facilitate the survival of resistant *Mtb* strains and impair the host’s immune response, creating a vicious cycle of treatment failure and disease progression (27). The persistence of dysbiosis, even after antibiotic cessation, underscores the need to investigate its long-term ramifications on TB management.

**Figure 2:**
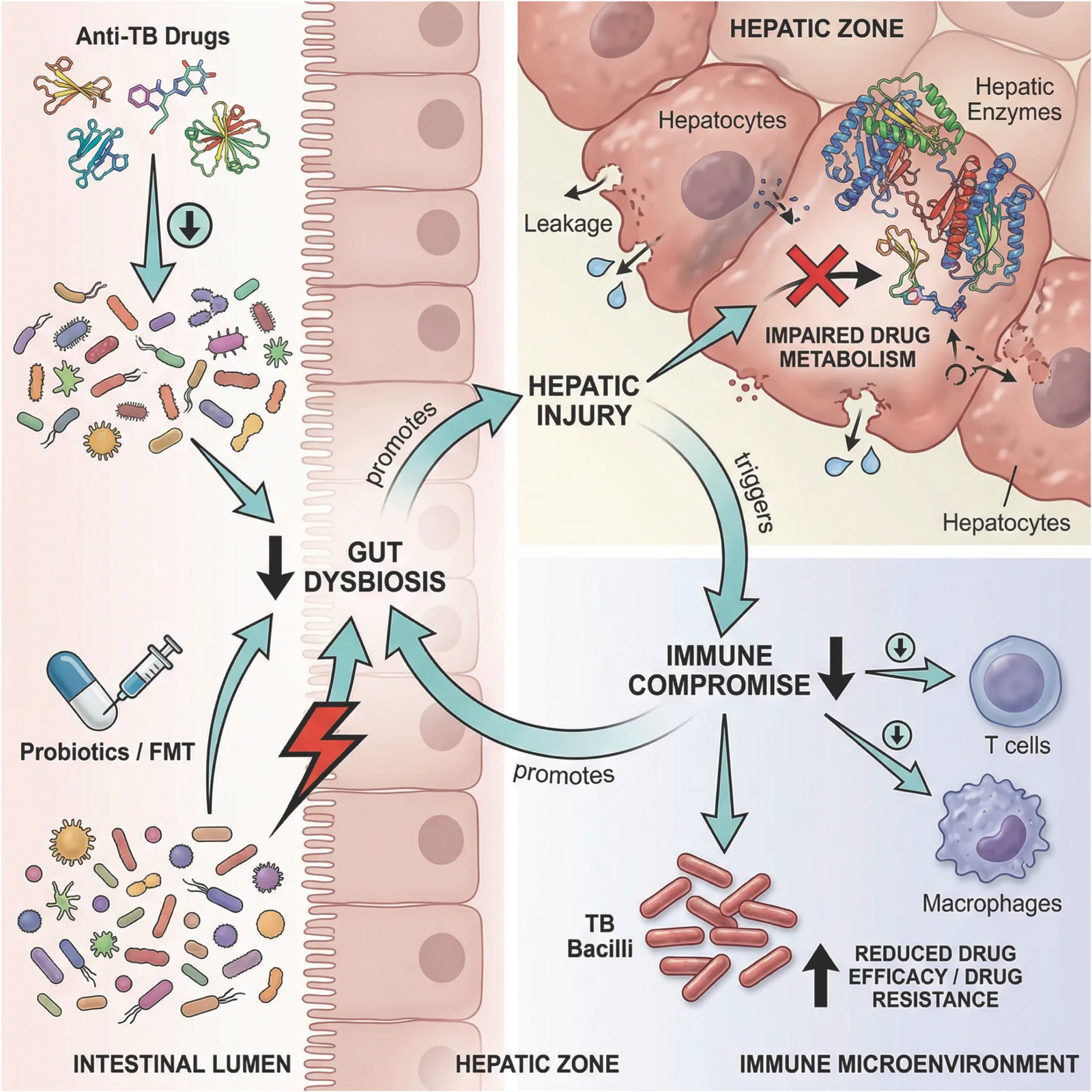
Mechanistic cascade of anti-tuberculosis drug-induced gut microbiota dysbiosis and clinical consequences. First-line anti-TB drugs induce gut dysbiosis, reducing microbial diversity and beneficial bacteria while elevating pathogens and impairing SCFA production. This drives intestinal barrier dysfunction and endotoxin (LPS) translocation to the liver via the gut-liver axis. Translocated endotoxins trigger ATB-DILI, characterized by inflammation, oxidative stress, and elevated aminotransferases (ALT/AST). Parallel immune dysregulation compromises *Mtb* clearance, promoting treatment failure, relapse, and MDR-TB. Probiotic *Bacteroides fragilis 839* counters this cascade by restoring microbial equilibrium, enhancing barrier integrity, suppressing inflammation, and augmenting immunity.

Collectively, anti-TB therapy initiates a detrimental triad: gut dysbiosis, hepatic injury, and blunted immune efficacy. These elements feed into a self-perpetuating cycle that drives treatment failure and fosters drug resistance (as illustrated in **Figure 2**). This detrimental triad—dysbiosis, hepatic injury, and immune blunting—feeds a vicious cycle that underscores gut health as a determinant of therapeutic success (28, 29). This cycle logically leads us to examine how this cycle manifests most severely in the context of DR-TB, where both the pathogen and the treatment pose an amplified threat to the microbiome.

Establishing a direct causal link between dysbiosis and the emergence of drug resistance is particularly challenging. Drug resistance is primarily driven by the genetic mutation of *Mtb* under antibiotic pressure. Although dysbiosis may create a permissive immunological environment, its effect is difficult to disentangle from the direct selective pressure exerted by prolonged, multi-drug therapy. Most studies reporting an association are retrospective and cannot rule out reverse causation, meaning that treatment failure itself may alter the microbiome.

## 4 The relationship between gut microbiota and drug-resistant tuberculosis and its metabolic characteristics

### 4.1 Structural changes of gut microbiota in patients with drug-resistant tuberculosis

DR-TB presents a compounded challenge: a resilient pathogen and prolonged, aggressive antibiotic regimens. This combined assault precipitates a distinct and severe gut microbiota derangement. Here, we characterize the specific structural and metabolic fingerprints of dysbiosis in MDR-TB, RR-TB, and pre-XDR-TB patients, arguing that these pathological shifts are key contributors to the persistent inflammation, poor treatment responses, and metabolic comorbidities observed in this population (28, 29).

The structural changes in gut microbiota are not only confined to the overall diversity but also involve specific shifts in the abundance of certain genera. A substantial increase in the abundance of *Enterococcus* and a concomitant decrease in *Lactobacillus* and *Bifidobacterium* have been observed in patients undergoing treatment for MDR-TB (26). These compositional shifts correlate with impaired immune responses, as the loss of beneficial bacteria that typically support gut health can lead to increased susceptibility to infections and poor treatment efficacy. The dynamic relationship between the gut microbiota and the host’s immune system is of paramount importance. Such dysbiosis can exacerbate inflammation and contribute to the systemic effects of tuberculosis (18). Furthermore, the long-term repercussions of DR-TB treatment on gut microbiota reveal that even after therapy ends, patients may persistently manifest dysbiosis, with substantial alterations persisting for years (29), which underscores the necessity for rigorous monitoring of intestinal health in TB patients, particularly those afflicted with drug-resistant forms of the disease. Understanding these alterations opens therapeutic possibilities, including the use of probiotics or FMT, to reestablish a healthy gut microbiome and enhance treatment outcomes.

Therefore, the gut microbiota in DR-TB is not merely disrupted; it is reprogrammed towards a pro-inflammatory, metabolically dysregulated state that fuels disease pathology and undermines therapy. Deciphering this reprogramming—particularly the functional output of microbial metabolites—is essential. This imperative raises a core question: how can we therapeutically intervene to correct this dysbiosis and break the cycle of treatment failure?

### 4.2 The association between gut microbiota dysbiosis and lipid metabolism and fatty acid synthesis

In patients with DR-TB, there is an increasing recognition of lipid metabolism abnormalities as a significant factor influencing disease progression and treatment outcomes. Gut microbiota dysbiosis contributes to the pathophysiology of various metabolic disorders, including those affecting lipid metabolism. The gut microbiota exerts a pivotal function in regulating lipid homeostasis and fatty acid synthesis through various mechanisms, including the modulation of host metabolic pathways and the production of microbial metabolites such as SCFAs (25). In patients with DR-TB, the alteration of gut microbiota composition may lead to increased levels of pro-inflammatory cytokines. These increased levels can exacerbate lipid accumulation in tissues and impair fatty acid oxidation processes. The presence of certain bacterial taxa, such as those from the *Firmicutes phylum*, is associated with elevated lipid synthesis and diminished fatty acid oxidation in the context of chronic inflammation (30). Furthermore, the gut microbiota influences the expression of genes involved in lipid metabolism, including those encoding fatty acid synthase (FASN) and acetyl-CoA carboxylase (ACC). The dysregulated lipid metabolism observed in DR-TB patients may thus be a consequence of both the inflammatory milieu created by the altered gut microbiota and the direct effects of specific microbial metabolites on host metabolic pathways (24). Such host-microbe interplay implies that therapeutic strategies restoring gut microbiota balance could ameliorate lipid metabolism disorders and improve clinical outcomes in DR-TB patients.

The regulation of fatty acid synthesis pathways by gut microbiota is central to disease progression in conditions such as tuberculosis, particularly in the context of drug resistance. Recent findings suggest that certain gut microbial populations may have the capacity to influence the host’s metabolic pathways, thereby impacting both lipid metabolism and the inflammatory response. Specifically, beneficial bacteria that augment the production of SCFAs can impede lipogenesis and promote fatty acid oxidation (31). In the context of tuberculosis, the pathogen *Mtb* utilizes host lipid metabolism for its survival and replication, particularly during latent infection stages. Dysbiosis can increase the availability of fatty acids for *Mtb*, thereby facilitating its growth and persistence within macrophages (32). Furthermore, the upregulation of fatty acid synthesis pathways, driven by alterations in the gut microbiota, exacerbates the inflammatory response, thereby further complicating the disease process. This is exemplified by the activation of pathways involving FASN and ACC in response to dysbiosis, which enhances lipid droplet formation in macrophages. These lipid droplets can then be utilized by *Mtb* for energy production (33). Consequently, this influence of gut microbiota on host metabolism and *Mtb* pathogenicity highlights the therapeutic potential of targeting the gut microbiota to restore metabolic balance and improve treatment outcomes.

### 4.3 The impact of gut microbiota metabolites on the pathology of drug-resistant tuberculosis

The complex interplay between gut microbiota and DR-TB is progressively acknowledged, particularly through the prism of microbial metabolites such as tryptophan (Trp) derivatives and SCFAs. Trp metabolism plays a pivotal role in modulating immune responses and maintaining gut homeostasis. Trp plays a dual role in the human body, who functions as a precursor for protein synthesis and as a substrate for the production of bioactive metabolites. These include kynurenine and indole derivatives, which have been shown to activate the AhR. This AhR activation is crucial, as it influences the differentiation and function of various immune cells, including T regulatory cells and dendritic cells, which are essential for maintaining immune tolerance and preventing excessive inflammation. In the context of DR-TB, where immune responses are frequently dysregulated, the metabolites derived from Trp metabolism may serve as potential therapeutic agents. The indole propionic acid (IPA), a metabolite produced by gut microbiota, has demonstrated antimicrobial activity against *Mtb* and may enhance host immune responses through its anti-inflammatory properties (34). Furthermore, SCFAs, which are primarily produced through the fermentation of dietary fibers by gut bacteria, have been shown to exert significant effects on immune modulation. These substances have been demonstrated to enhance the production of anti-inflammatory cytokines and to inhibit the release of pro-inflammatory mediators. Consequently, they may potentially counteract the inflammatory processes associated with DR-TB. Research has demonstrated that SCFAs can influence the expression of genes involved in immune responses, thereby playing a protective role against infections, including tuberculosis (35). This metabolite-immune axis manifests as clinically observable pathological patterns in DR-TB patients, with specific microbial molecules demonstrating distinct immunoregulatory capacities and lesion-modifying effects, as systematically categorized in **Table 1**.

**Table 1:** Clinically validated microbial metabolites in DR-TB pathology.

Metabolite category	Key molecules	Biosynthetic pathway	Immunomodulatory action	Impact on DR-TB pathology
SCFAs (24, 35)	Butyrate, Propionate, Acetate	Dietary fiber fermentation by *Bacteroides*, *Faecalibacterium*	• HDAC inhibition • ↑ IL-10 production • ↑ Treg differentiation	• ↓ Lung cavitation severity • ↓ Granulomatous necrosis
Tryptophan Derivatives (34)	Indole propionic acid (IPA)	Tryptophan metabolism by *Bacteroides*, *Clostridium*	• AhR activation • PPARγ pathway modulation • ↓ NF-κB signaling	• ↑ Sputum conversion rate • ↓ Caseous necrosis formation
Lactobacillus-mediated metabolism (18)	Kynurenine	• AhR ligand delivery • IDO1 enzyme modulation	• AhR ligand binding → Treg/Th17 balance • ↓ Inflammasome activation • IDO1 pathway modulation	• ↑ Extrapulmonary dissemination risk • ↑ Pleural effusion severity
Fatty Acid Mediators (33)	Conjugated linoleic acid (CLA)	Linoleic acid metabolism by *Bifidobacterium*	• PPARα activation • ↓ FASN expression	• Inhibits *Mtb* lipid droplet formation • Promotes bacterial clearance
Specialized Pro-resolvers (30, 32)	Resolvin D1	*Bifidobacterium* ω-3 metabolism	• ↑ Efferocytosis in macrophages • ↓ Neutrophil infiltration • ↓ IL-1β/IL-6 production • ↑ TGF-β signaling	• ↓ Cavitary lesion progression • ↓ TB reactivation risk

The symbol ↑ represents an upward adjustment, while the symbol ↓ represents a downward adjustment or diminution.

The dynamic relationship between the aforementioned metabolites and immune cell functionality is of paramount importance. SCFAs have been demonstrated to facilitate the differentiation of T helper cells, which are essential for orchestrating the immune response against *Mtb*. Furthermore, the alterations in the composition of the gut microbiota, which are frequently observed in patients with DR-TB, can result in a reduction in the production of these beneficial metabolites, thereby further exacerbating the disease pathology (4). This dysbiosis impairs the host’s ability to mount an effective immune response and may also facilitate the persistence and proliferation of drug-resistant strains of *Mtb*. Consequently, elucidating the metabolic pathways associated with gut microbiota and their impact on immune modulation offers a promising approach for developing novel therapeutic strategies aimed at restoring gut health and enhancing host defenses against DR-TB. We hypothesize that targeting the gut-lung axis through dietary interventions or probiotics that promote the production of beneficial metabolites, treatment outcomes may be improved and the burden of drug-resistant tuberculosis may be reduced (36).

## 5 The application potential of gut microbiota regulation strategies in tuberculosis treatment

### 5.1 Probiotics and their therapeutic potential

Recognizing the gut microbiota as a driver of TB pathology and treatment complications logically pivots the focus to therapeutic modulation. This section critically evaluates the arsenal of microbiota-targeted strategies, from direct supplementation (probiotics) and ecosystem replacement (FMT) to dietary and prebiotic support. We assess their mechanistic rationale, current evidence, and potential to act as force multipliers for conventional anti-TB therapy.

Probiotics, specifically strains such as *B. fragilis 839*, have garnered attention for their potential to mitigate the adverse effects associated with anti-tuberculosis drug therapy. The adverse effects of anti-tuberculosis medications, including hepatotoxicity and gastrointestinal disturbances, significantly hinder treatment adherence and overall patient outcomes. Probiotic supplementation mitigates these adverse effects by restoring gut microbiota balance and enhancing intestinal barrier function. For example, *B. fragilis* modulates host immune responses, reducing inflammation and promoting intestinal homeostasis. This immunomodulatory capacity may directly counteract the adverse effects of anti-tuberculosis drugs.

Furthermore, the immunomodulatory properties of probiotics contribute to their therapeutic potential in the treatment of tuberculosis. Probiotics such as *B. fragilis 839* have been shown to stimulate the production of specific cytokines, thereby enhancing the host’s immune response against *Mtb*. The dual action of probiotics, functioning as both a protective agent against drug-induced side effects and an enhancer of immune function, positions them as a promising adjunctive therapy in the management of tuberculosis. Recent research has highlighted that the incorporation of probiotics into treatment regimens not only helps in managing the adverse effects of anti-tuberculosis drugs but also may improve the overall efficacy of the treatment by fostering a more favorable gut microbiota composition (35). However, findings from other infectious disease contexts have been mixed, with some RCTs failing to demonstrate a significant clinical benefit of probiotics. This inconsistency highlights the need for TB-specific clinical trials to validate their therapeutic potential.

The therapeutic potential of probiotics, particularly in the context of tuberculosis treatment, is multifaceted. These agents offer a dual benefit: they alleviate the side effects of anti-tuberculosis medications while simultaneously enhancing the immune response against the infection. This renders probiotics a valuable consideration in the development of comprehensive treatment strategies for tuberculosis, especially in light of the increasing prevalence of drug-resistant strains and the need for effective adjunctive therapies. Future clinical studies are warranted to further elucidate the mechanisms by which probiotics exert their effects and to establish standardized protocols for their use in conjunction with anti-tuberculosis therapies (37).

Despite promising preclinical data, the clinical evidence for probiotics in TB is scarce and preliminary. Key challenges include: (1) Strain-specificity: The beneficial effects observed with *B. fragilis* 839 in animal models may not be generalizable to other probiotic strains. (2) Safety in immunocompromised hosts: TB patients, especially those with advanced disease or HIV co-infection, may be at risk of probiotic-associated infections. (3) Regulatory and quality control issues: The lack of stringent regulation for probiotics as dietary supplements leads to variability in product composition and viability. Rigorous, placebo-controlled randomized clinical trials are urgently needed to establish efficacy, optimal dosing, and long-term safety in TB patients.

### 5.2 Exploration of fecal microbiota transplantation in tuberculosis

FMT has emerged as a promising therapeutic strategy in the management of various diseases, including TB. The theoretical underpinnings of FMT’s role in regulating gut microbiota balance and immune response are anchored in the concept of the gut-lung axis, which posits that gut microbiota exerts a substantial influence on systemic immunity. Dysbiosis, or the imbalance of gut microbiota, has been implicated in the pathogenesis of TB, as it can compromise immune functions that are critical for controlling *Mtb* infection. A healthy gut microbiome contributes to the maintenance of immune homeostasis, which is essential for effective immune responses against *Mtb*. Specifically, gut microbiota-derived metabolites, such as SCFAs, have been shown to enhance the production of anti-inflammatory cytokines, thereby promoting a robust immune response against TB. In addition, FMT can reinstate a healthy intestinal environment, thereby fortifying the host’s defenses against bacterial infections, including *Mtb*, by regulating immune pathways and enhancing intestinal barrier function (16, 38).

Preliminary clinical and animal studies investigating the potential application of FMT in treating TB and related immunological diseases have yielded encouraging results. In animal models, FMT has been observed to restore gut microbiota diversity, which is often diminished in TB-infected hosts. Restoring microbiota diversity through FMT has been linked to enhanced immune responses, including elevated phagocytic activity of macrophages and augmented production of *Mtb*-killing cytokines, such as IFN-γ and TNF-α. Recent clinical studies have begun to investigate the potential of FMT in humans with TB, particularly in cases where conventional therapies have failed or where patients exhibit significant dysbiosis. Preliminary findings suggest that FMT can lead to a reduction in TB-related symptoms and may improve treatment outcomes by enhancing the immune response to *Mtb*. However, given the preliminary nature of this evidence, large-scale, well-designed clinical trials are urgently needed to establish the efficacy and safety of FMT in TB patients (39, 40).

FMT’s potential is particularly noteworthy in addressing a key consequence of TB therapy: the excessive use of antibiotics in the treatment of TB induces substantial alterations in the composition of the gut microbiota, thereby engendering an environment conducive to the proliferation of drug-resistant strains of *Mtb*. The restoration of a balanced gut microbiome through FMT has the potential to mitigate the adverse effects of antibiotic treatment, thereby reducing the risk of drug resistance. Furthermore, FMT has the potential to enhance the efficacy of anti-TB drugs by improving the overall immune response and reducing inflammation associated with dysbiosis. Nevertheless, the implementation of FMT as a standard treatment option for TB must be approached with caution due to potential risks, including the transmission of pathogens through fecal material. A comprehensive screening of fecal samples from donors, in conjunction with rigorous clinical protocols, will be essential to ensure patient safety (41, 42). Current studies on gut microbiota and TB exhibit high methodological heterogeneity and small sample sizes, a limitation noted in a recent meta-analysis that underscores the need for standardized protocols and larger cohorts.

FMT is a novel and promising therapeutic approach in the management of tuberculosis. It has the potential to restore gut microbiota balance, enhance immune responses, and reduce the risks of drug resistance and recurrence. Preliminary studies suggest that FMT may yield positive outcomes; however, further research is necessary to establish standardized protocols, assess long-term effects, and evaluate the overall safety of FMT in patients with TB. The incorporation of FMT into TB treatment regimens has the potential to transform the management of this persistent global health challenge (43, 44).

While FMT holds theoretical promise for restoring a healthy ecosystem, its application in TB is fraught with hurdles. (1) Donor screening: Rigorous screening for pathogens, including multi-drug resistant organisms and parasites, is essential but logistically complex and costly. (2) Route of administration and durability: The optimal route (e.g., oral capsules vs. colonoscopy) and the durability of engraftment in patients on concurrent antibiotics are unknown. (3) Risk of adverse events: Beyond pathogen transmission, FMT carries risks of inducing inflammatory bowel disease or metabolic syndrome in recipients. (4) Lack of standardized protocols: There are no established protocols for FMT in TB, and its use should be confined to well-designed clinical trials with stringent safety monitoring. The gap between mechanistic promise and clinical implementation remains substantial.

### 5.3 Other microecological intervention strategies

The modifiable relationship between gut microbiota and TB is a growing focus of research, particularly the influence of dietary interventions and microbial metabolites on disease outcomes. Prebiotics, non-digestible food components that stimulate the growth of beneficial gut bacteria, have demonstrated efficacy in modulating the gut microbiota composition and enhancing the immune response against pathogens like *Mtb*. Studies have indicated that dietary fibers can lead to an increase in SCFAs, which are known to exert anti-inflammatory effects and enhance gut barrier function (45). In addition, the supplementation of particular microbial metabolites, such as butyrate, has been associated with enhanced immune responses and decreased inflammation. This suggests that dietary interventions could function as complementary therapeutic modalities in the management of tuberculosis (46). Furthermore, the potential of probiotics in restoring microbiota balance in patients with tuberculosis has been emphasized, where probiotics not only facilitate the re-establishment of gut flora but also potentially enhance the efficacy of conventional TB therapies (47). The incorporation of prebiotics and probiotics into dietary regimens could thus provide a multifaceted approach to TB treatment, targeting both the microbiome and the host’s immune system. The translational promise of microbiota-targeted strategies is further crystallized in **Table 2**, which synthesizes mechanistic insights and clinical outcomes across probiotic, FMT, and synergistic intervention approaches for comprehensive TB management.

**Table 2:** Preclinical and clinical evidence for microbiota-targeted interventions in TB management.

Intervention type	Key agents/approaches	Mechanism of action	TB-specific effects	Evidence Level
Probiotics (6)	• *Bacteroides fragilis 839* • *Lactobacillus casei* • *Bifidobacterium longum*	• Restoration of microbiota balance • Intestinal barrier enhancement • SCFA production ↑ • Anti-inflammatory cytokine induction	• ↓ Hepatotoxicity • ↑ Rifampicin bioavailability • ↓ Treatment interruption rates • ↑ Sputum conversion speed	Preclinical (animal/*in vitro*) and Human observational study
FMT (16, 38)	• Healthy donor microbiome • Aerobic/anaerobic processed material	• Microbial diversity restoration • Gut-lung axis modulation • Treg/Th17 rebalancing • Butyrate-producing bacteria ↑	• ↓ Cavitation progression • XDR-TB relapse ↓ • Isoniazid metabolism optimization • ↑ Granuloma organization	Preclinical (animal/*in vitro*) and Human observational study
Prebiotics & Metabolites (45, 46)	• Galacto-oligosaccharides • Resistant starch • Butyrate supplements	• Selective growth of commensals • HDAC inhibition • GPCR activation (GPR43/109a) • M2 macrophage polarization	• ↓ Extrapulmonary dissemination • ↓ Pleural effusion volume • Lung fibrosis ↓ • Cavity wall thickness ↓	Preclinical (animal/*in vitro*)
Synbiotic formulations (47)	• *B. fragilis* + Inulin • *L. reuteri* + FOS	• Cross-feeding networks • Sustained SCFA release • Pathogen exclusion • AhR ligand production	• ↓ MDR-TB emergence • Drug-induced dysbiosis ↓ • Treatment completion ↑ • Immune reconstitution	Preclinical (animal/*in vitro*)

The symbol ↑ represents an upward adjustment, while the symbol ↓ represents a downward adjustment or diminution.

**Figure 3:**
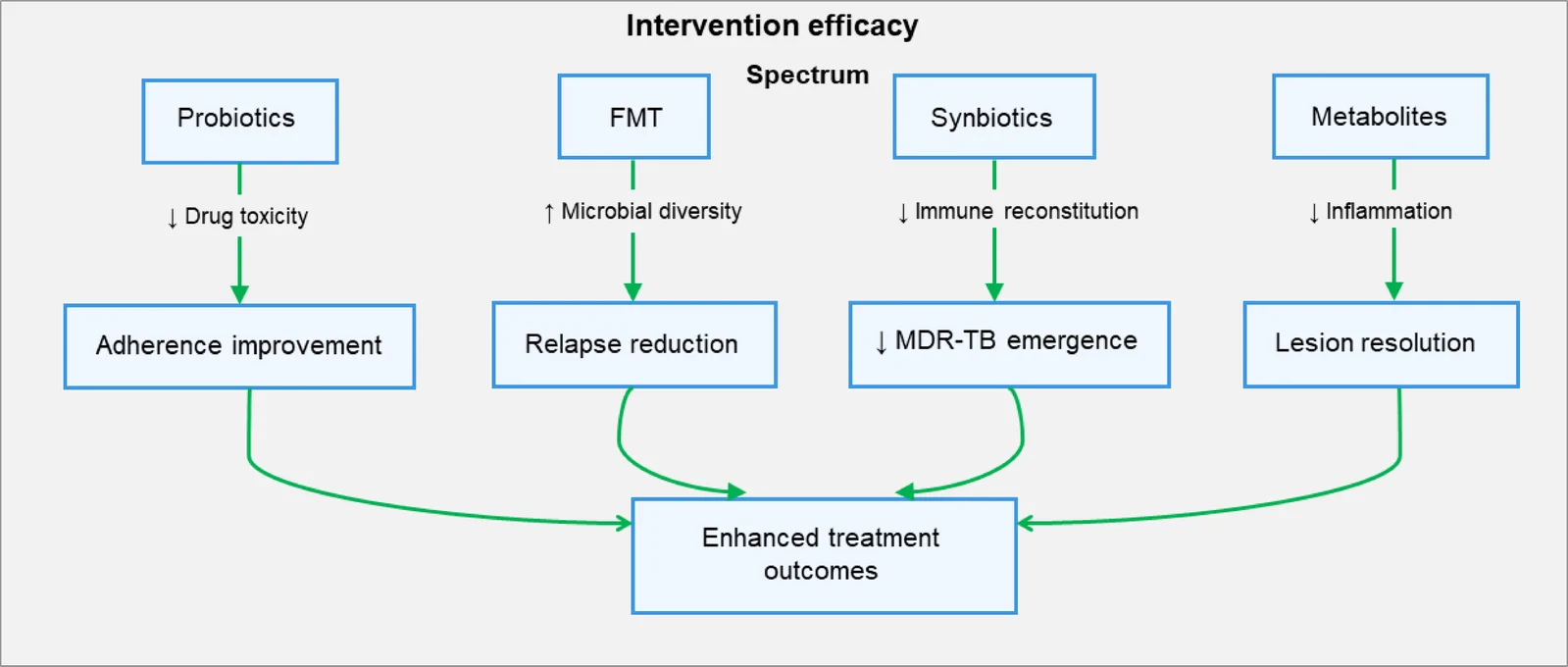
Mechanistic integration of microbiota interventions in tuberculosis treatment. This schematic illustrates how probiotics, FMT, synbiotics, and microbial metabolites employ complementary mechanisms, such as toxicity mitigation, diversity restoration, immune potentiation, and pathological resolution, to enhance tuberculosis treatment outcomes collectively by modulating the microbiome-immune network. The symbol ↑ represents an upward adjustment, while the symbol ↓ represents a downward adjustment or diminution.

As synthesized in **Figure 3**, diverse microbiota-targeted interventions converge on a common therapeutic goal: to bolster the host ecosystem. They achieve this through complementary biological mechanisms that collectively mitigate drug toxicity, prevent resistance, resolve inflammation, and ultimately elevate treatment success rates. Integrating these microecological interventions (e.g., prebiotics, probiotics) with conventional TB therapy thus represents a potent strategy to enhance outcomes, as evidenced by emerging research. For instance, co-administration of *Lactobacillus casei* with anti-TB drugs has been shown to mitigate gastrointestinal adverse effects and improve gut microbiota profiles, demonstrating a direct protective effect against antibiotic-induced dysbiosis (45). Such synergy—for example, combining dietary interventions that yield bioactive microbial metabolites with standard drugs—can enhance drug efficacy by modulating metabolism and bioavailability (46). This host-microbe co-management strategy is especially promising for high-risk populations, such as patients with TB and diabetes, where underlying dysbiosis may otherwise compromise treatment adherence and outcomes (47). In summary, the integrated application of microbiota-targeted and conventional therapies marks a transformative direction in TB care. By concurrently targeting the pathogen and fortifying the host’s microbial ecosystem, this paradigm moves beyond mere pathogen eradication towards sustainable cure and improved quality of life.

## 6 Interaction between new anti-tuberculosis drugs and gut microbiota

### 6.1 The impact of novel drugs on gut microbiota and resistance risks

The future of anti-TB drug development lies not only in killing *Mtb* more effectively but also in preserving the host’s microbial ally. This chapter explores this paradigm shift, focusing on two frontiers: 1) the design of narrow-spectrum drugs that minimize collateral damage to the gut microbiome, and 2) the exploitation of gut microbiota metabolites as novel anti-infective agents or adjuncts.

The advent of narrow-spectrum anti-TB drugs, such as benzofuroxan derivatives, has been a significant advancement in the fight against TB and MDR-TB. These narrow-spectrum agents are designed to minimize disruption of the gut microbiota, a pivotal factor in sustaining global health and forestalling secondary infections. Research indicates that traditional broad-spectrum antibiotics can lead to dysbiosis, characterized by a reduction in microbial diversity and the emergence of antibiotic-resistant strains (48). By preserving beneficial gut bacteria, narrow-spectrum agents help maintain colonization resistance, thereby reducing the risk of antibiotic-associated complications such as *Clostridioides difficile* infection (49). The protective role of gut microbiota is further underscored by studies showing that alterations in microbial composition can influence drug metabolism and efficacy, highlighting the need for drug designs that consider microbiota preservation (50). This microbiota-preserving design strategy can enhance therapeutic outcomes of anti-TB treatments and mitigate the risk of developing resistance, a critical concern in TB management.

This recognition of the need to preserve gut microbiota has spurred drug development strategies that explicitly prioritize microbiome protection. The incorporation of microbiome considerations into drug design, by incorporating adjunctive probiotics or prebiotics, can foster the development of therapies that are not only effective against pathogens but also actively support and enhance the resilience of the host’s microbiota during anti-TB treatment (51, 52). These strategies are designed to create a synergistic effect, whereby the pharmacological action of the drug is complemented by the microbiota’s role in enhancing immune responses and reducing inflammation (53). Furthermore, the exploration of microbial metabolites as biomarkers for treatment efficacy and resistance profiles is an emerging field with the potential to transform the approach to TB therapy (50).

The development of novel anti-TB drugs that consider the impact on gut microbiota represents a paradigm shift in the treatment of infectious diseases. By concentrating on narrow-spectrum agents and incorporating microbiota-protective strategies, researchers can enhance treatment efficacy while minimizing the risks associated with antibiotic resistance and dysbiosis. This dual-pronged approach—combining targeted antimicrobial action with microbiome preservation not only addresses the pressing challenges of TB but also establishes a foundation for future drug development across a range of therapeutic areas, underscoring the pivotal role of the gut microbiome in health and disease management (49).

### 6.2 Gut microbiota metabolites as auxiliary targets for anti-tuberculosis drugs

The role of gut microbiota metabolites, particularly indole propionic acid (IPA), in inhibiting *Mtb* has garnered significant attention in recent research. Emerging evidence points to the promising anti-tubercular properties of IPA, a byproduct of gut microbial metabolism. This natural substance has shown the capacity to impede the proliferation of *Mtb*, highlighting its potential as a therapeutic agent in combating tuberculosis. While its capacity for direct bacterial inhibition is still being elucidated, its primary therapeutic potential may lie in immunomodulation and mitigating inflammation (34). The therapeutic potential of IPA is further underscored by its detection in the serum of both healthy individuals and tuberculosis patients, suggesting that gut microbiota may influence susceptibility to mycobacterial diseases (34). This observation highlights the significance of the gut-lung axis, wherein gut microbiota-derived metabolites can serve as both biomarkers for disease progression and prospective therapeutic agents. The exploration of IPA and analogous metabolites has revealed potential avenues for the development of microbiota-based therapies that could complement conventional anti-tuberculosis treatments, particularly in populations where inflammation exacerbates disease morbidity and mortality.

The potential for leveraging gut microbiota metabolic pathways to identify novel anti-tuberculosis drugs is a promising avenue for further research. In light of the escalating prevalence of drug-resistant strains of *Mtb*, there is an imperative for the development of innovative therapeutic strategies. Studies have indicated that targeting specific metabolic pathways associated with gut microbiota could lead to the identification of new drug candidates with anti-tubercular activity. Modulating pathways involved in the synthesis of SCFAs and other metabolites represents a promising strategy to influence the immune response and enhance host resistance to infections. Moreover, network pharmacology methodologies enable researchers to elucidate the intricate relationships between gut microbiota, their metabolites, and host immune targets, thereby facilitating a more comprehensive understanding of how these interactions can be harnessed for therapeutic purposes (54). These network pharmacology-based methodologies not only facilitates the identification of novel compounds but also aids in understanding the mechanisms through which these metabolites exert their effects, thereby providing a solid foundation for the development of new anti-tuberculosis drugs. The potential for gut microbiota metabolites to serve as auxiliary targets in tuberculosis treatment represents a significant advancement in the quest for effective therapies, particularly in the context of rising antibiotic resistance and the need for multifaceted treatment strategies.

### 6.3 Application of computational biology in the discovery of anti-tuberculosis drug targets

The application of computational biology to anti-TB drug target discovery represents a paradigm shift in our approach to combating *Mtb* and its resistant strains. A particularly salient avenue within this domain entails the employment of proteomics for the identification of pathogen-specific targets, a strategy that serves to mitigate the repercussions on the gut microbiota. By leveraging computational tools, researchers can construct comprehensive protein-protein interaction (PPI) networks that elucidate the biological functions and interactions of proteins within *Mtb*. Building comprehensive PPI networks not only facilitates the identification of potential drug targets but also deepens our understanding of their structural biology, a process imperative for targeted small-molecule modulator development (55). Computational methods can streamline the drug discovery process by predicting binding affinities and stability between drug candidates and target proteins, thereby enhancing screening efficiency (56). Moreover, integrating cheminformatics and molecular dynamics simulations explores resistance mechanisms, providing insights into how certain proteins contribute to survival and virulence (57). This integrated computational strategy thus enables a dual benefit: identifying novel targets and facilitating the design of drugs that inhibit them while sparing the gut microbiome.

Furthermore, targeting the virulence factors of *Mtb* through anti-virulence therapies is a novel strategy, offering additional benefits to the gut microbiota. By concentrating on the mechanisms that enable the pathogen to evade the host immune response, researchers can develop therapies that do not directly kill the bacteria but instead disarm them, thereby reducing the selective pressure for resistance (58). Anti-virulence therapy is particularly salient in the context of rising drug-resistant tuberculosis, where conventional antibiotics may prove ineffective. Inhibitors that target specific enzymes or pathways critical for the pathogen’s identifying such targets through computational methods—including high-throughput virtual screening—enables the rapid assessment of potential inhibitors and their interactions with *Mtb* proteins, thereby facilitating the discovery of compounds that disrupt the pathogen’s lifecycle while preserving the gut microbiome (59).

From augmenting current regimens with probiotics to designing next-generation, microbiome-sparing drugs, the translational pipeline is advancing. These strategies collectively move TB management towards a holistic framework that co-manages the infection and the host’s microbial ecosystem. However, a comprehensive patient-care model must also address the less visible wounds of TB—the psychological toll, which itself may be rooted in the gut-brain axis disruptions caused by disease and treatment.

## 7 The impact of gut microbiota dysbiosis on the mental health of tuberculosis patients

### 7.1 The mechanism of antituberculosis drugs on the gut-brain axis

The burden of TB extends beyond physical morbidity to encompass significant mental health sequelae, including depression and anxiety. Emerging evidence implicates the gut-brain axis as a critical link, wherein anti-TB drug-induced dysbiosis disrupts the production of neuroactive metabolites and promotes neuroinflammation, contributing to mood disorders (60).

The role of the brain-gut-microbiota axis extends beyond mere mood regulation; it also encompasses the modulation of immune responses and inflammation. Dysbiosis resulting from antibiotic treatment has been demonstrated to lead to an altered immune response, which may exacerbate neuroinflammation and contribute to psychiatric symptoms. A body of research has indicated that antibiotics can lead to increased levels of inflammatory cytokines, which have been implicated in the pathogenesis of depression and anxiety disorders (61). The relationship between gut microbiota and the central nervous system (CNS) is intricate, with evidence indicating that gut-derived signals can influence brain function and behavior through various pathways, including the vagus nerve and systemic circulation. These mechanistic insights into the gut-brain axis highlight the promise of adjunctive, microbiota-targeted strategies to alleviate psychiatric symptoms in patients on anti-TB therapy. Interventions such as probiotics or dietary modifications aimed at restoring gut microbiota diversity could serve as adjunct therapies to improve mental health outcomes in these patients, thereby enhancing their overall treatment experience and adherence to antituberculosis regimens.

The impact of antibiotics on the gut-brain axis is multifaceted, with significant implications for mental health. The decrease in gut microbiota diversity due to antibiotic therapy can lead to adverse psychological outcomes, emphasizing the need for a holistic approach that considers both physical and mental health. Future research should elucidate the specific mechanisms by which gut microbiota influence mental health and explore potential interventions to mitigate the negative effects of antibiotics on the gut-brain axis in TB patients (22).

### 7.2 The potential impact of gut microbiota regulation on the psychological state of tuberculosis patients

The dynamic relationship between gut microbiota composition and mental health is receiving growing attention, particularly in the context of chronic diseases such as TB. Probiotics and microbiota-based therapies are emerging as potential interventions to mitigate psychological symptoms associated with TB. Mechanistically, these beneficial bacteria may influence the gut-brain axis, where gut microbiota can produce neurotransmitters and metabolites that modulate mood and cognitive function. SCFAs, produced by gut bacteria, exert neuroprotective properties and stimulate the production of brain-derived neurotrophic factor (BDNF), which is essential for neuronal health and cognitive function (62). Thus, interventions that boost SCFA production may directly support neural health in TB patients. Additionally, the regulation of inflammatory pathways by gut microbiota may also contribute to mental health, as chronic inflammation has been associated with mood disorders. In patients diagnosed with TB, the dysbiosis that is frequently observed in cases of prolonged antibiotic treatment has been shown to exacerbate psychological distress, manifesting in symptoms such as anxiety and depression (60). Consequently, restoring gut microbiota balance through probiotics or dietary interventions can alleviate these mental health issues, providing a dual benefit of improving both physical and psychological outcomes in TB management.

Psychological health management is a critical and often underappreciated component of comprehensive TB care. Mental health issues, including depression and anxiety, are prevalent among TB patients and can significantly hinder treatment adherence and overall recovery (63). The social disapproval associated with TB, in conjunction with the physiological demands of the disease, frequently intensifies feelings of isolation and despair among patients. Integrating mental health services into TB care is imperative. This integration should include routine screening for psychological distress and the provision of appropriate interventions, such as counseling and psychiatric support, alongside standard TB treatment. Furthermore, healthcare providers should undergo training to recognize and address mental health needs, thereby fostering a holistic approach to patient care that encompasses both physical and psychological well-being (64). By prioritizing mental health, healthcare systems can improve treatment outcomes and enhance the quality of life for TB patients, ultimately contributing to more effective public health strategies in combating this global health crisis.

### 7.3 Clinical practice focus on the mental side effects of anti-tuberculosis treatment

The treatment of TB is often accompanied by significant challenges, particularly with respect to the mental health of patients undergoing anti-TB therapy. The psychiatric side effects associated with anti-TB medications have been shown to have a significant impact on treatment adherence and overall patient well-being. The implementation of effective monitoring and intervention strategies is imperative in addressing these mental health concerns. A substantial body of research suggests a link between the administration of antibiotics, including those used to treat TB, and the occurrence of psychiatric side effects, such as depression and anxiety. The association is primarily attributed to the brain-gut-microbiota axis, which serves as a crucial mediator (60). This gut-brain-microbiota interplay indicates that the gut microbiota, influenced by antibiotic treatment, can modify mental health outcomes. Dysbiosis has been linked to increased susceptibility to psychiatric disorders, including major depressive disorder (65). Clinicians must exercise vigilance in monitoring patients for indications of mental health deterioration during treatment, as a considerable proportion of patients with DR-TB experience psychological distress. Research has indicated that up to 62.7% of patients may present with depressive symptoms (66). Effective management should encompass: routine mental health screening, timely referral to specialists, and integration of supportive therapies addressing both physical and psychological aspects of TB.

Therefore, the psychological distress in TB patients is not merely a reactive psychosocial phenomenon but is biologically rooted in therapy-induced gut dysbiosis. Addressing mental health is thus an integral component of comprehensive care and can be advanced through microbiota-supportive interventions. Adopting this holistic perspective—encompassing the lung, gut, liver, and brain—underscores the overarching conclusion for an integrated fight against TB.

## 8 Conclusion

The burgeoning field of gut microbiota research has fundamentally reshaped our understanding of TB. This review synthesizes compelling evidence to recast the gut microbiome from a passive bystander to a central regulator in a multidirectional dialogue encompassing TB susceptibility, treatment efficacy, drug toxicity, and the emergence of resistance. We have elucidated a pathogenic cycle wherein first-line anti-TB therapies, while targeting *Mtb*, concurrently induce profound gut dysbiosis. This dysbiosis subsequently compromises host immunity via the gut-lung axis, exacerbates drug-induced liver injury, and potentially fosters an environment conducive to drug-resistant strains. Consequently, gut health emerges as a critical determinant of therapeutic success, inextricably linking microbiotal equilibrium to clinical outcomes.

This paradigm shift—from a purely pathogen-centric to a holistic host-microbe co-management strategy—necessitates a fundamental reevaluation of TB therapeutics. It is crucial to note that while the gut-lung axis and the dysbiosis-driven model are compelling, the majority of evidence supporting direct causality comes from animal models. Human studies are largely correlative, and definitive proof of causation in patients remains a key challenge for the field. Promisingly, microbiota-targeted interventions such as specific probiotics and FMT offer tangible avenues to restore homeostasis, mitigate adverse effects, and potentially augment cure rates. Concurrently, the next generation of anti-TB drug development must prioritize “microbiome-sparing” designs, such as narrow-spectrum agents or therapies leveraging beneficial microbial metabolites like indole propionic acid.

Furthermore, the gut-brain axis compels the recognition that comprehensive TB care must address the psychological sequelae of the disease and its treatment, integrating mental health support into standard management protocols. Looking forward, key challenges and opportunities define the research agenda: 1) Deepen mechanistic understanding by unraveling the precise molecular signals that mediate the gut-lung and gut-brain axes in TB. 2) Discover biomarkers by identifying gut microbial or metabolomic signatures that predict individual risk for drug toxicity, treatment failure, or depression. 3) Translate to the clinic through large-scale, rigorous RCTs to establish the efficacy, safety, and protocols for probiotics and FMT in diverse TB patient populations. 4) Advance personalized medicine by integrating microbiome data with host genetics and clinical parameters to develop tailored therapeutic strategies.

In conclusion, to successfully confront the enduring threat of TB—especially its drug-resistant forms—requires embracing the complexity of the host ecosystem. By forging collaborative links across microbiology, immunology, pharmacology, and psychiatry, we can advance integrated strategies that simultaneously disarm the pathogen and fortify the host. This holistic, host-microbe co-management approach offers a path to not only overcome TB but also to pioneer a more sustainable and patient-centric paradigm for infectious disease management. It is also important to acknowledge that not all studies have found strong associations. Some research suggests that the gut microbiome changes observed in TB patients may be largely driven by confounding factors such as hospitalization, antibiotic co-exposure, and dietary changes rather than the disease itself. Future studies with rigorous longitudinal designs are needed to resolve these discrepancies.
